# Letter to the editor in response to “Rare presentation of lues maligna and nodular syphilis in an immunocompetent patient”

**DOI:** 10.1016/j.jdcr.2026.03.068

**Published:** 2026-04-30

**Authors:** Tarun Narang, Sejal Jain, Bhushan Kumar

**Affiliations:** a Department of Dermatology, Venereology and Leprology, Postgraduate Institute of Medical Education and Research, Chandigarh, India; b Department of Dermatology, Shalby Hospital, SAS Nagar, Punjab, India

**Keywords:** immunosuppression, lues maligna, nodular syphilis, penicillin, secondary syphilis

*To the Editor*:We read with interest the case report by Al-Abdulla et al^1^ entitled “Rare presentation of lues maligna and nodular syphilis in an immunocompetent patient” published in *JAAD Case Reports*. While the clinical, dermoscopic, serologic, and histopathologic findings support secondary syphilis (SS), we question the classification as lues maligna (LM), the treatment regimen, and certain other interpretations.

LM is a rare ulceronodular variant of SS characterised by rapidly progressive painful, necrotic “punched-out” ulcers with dark rupioid crusts and erythematous halos, often associated with immunosuppression.^2^ The distinction between LM and SS is not based on clinical features alone, but requires correlation with histopathologic and systemic findings. Diagnostic criteria for LM include severe ulceronodular lesions with constitutional symptoms, high Venereal Disease Research Laboratory test titers, characteristic histopathology, and an increased frequency of intense Jarisch-Herxheimer reaction.^3^ In the present case, lesions were erythematous papulonodules without rapid ulceration, pain, necrosis, or rupioid crusting. Pruritus with pityriasis rosea-like collarette scales aligns more closely with papulo-squamous SS. The intermittent fever may have been incidental. Consideration of papular urticaria and papular pityriasis rosea as differentials further confounds the distinction. Dermoscopy revealed Biett’s collarette with dotted and glomerular vessels, features of papulosquamous SS, without rupioid crusts of LM.^4^ Histopathology showed plasma cells and loose granulomas typical of SS but lacked obliterative endarteritis, fibrinoid vasculitis, or ischemic necrosis expected in LM.^3^

The patient received 3 weekly benzathine penicillin G doses (2.4 million units). The Centers for Disease Control and Prevention recommends 1 dose for primary, secondary, and early latent syphilis; 3 doses are reserved for the late latent stage or syphilis of unknown duration.^5^ One dose of benzathine penicillin, therefore, would have sufficed in this patient. Follow-up details would have strengthened the report.

We suggest reclassification as papulonodular SS to align with established criteria, and adhering to treatment guidelines to avoid overtreatment.

## Conflicts of interest

None disclosed.
